# Low handgrip strength in advanced cancer: associated factors and clinical implications in palliative care

**DOI:** 10.1007/s00520-026-11249-x

**Published:** 2026-09-26

**Authors:** Larissa Pereira Santos, Bianca Sakamoto Ribeiro Paiva, Karla Santos Da Costa Rosa, Carlos Eduardo Paiva, Livia Costa De Oliveira

**Affiliations:** 1 GPQual—Palliative Care and Quality of Life Research Group, Barretos Cancer Hospital, São Paulo, Brazil; 2 GP-ECPal—Cancer Epidemiology and Palliative Care Research Group, National Cancer Institute (INCA), 274 Visconde de Santa Isabel, Rio de Janeiro, RJ 20560-120 Brazil

**Keywords:** Advanced cancer, Palliative care, Associated factors in functional capacity, Handgrip strength

## Abstract

**Introduction:**

Handgrip strength (HGS) is a widely used clinical and prognostic tool in oncology and is associated with several outcomes in palliative care (PC).

**Objective:**

To evaluate the factors associated with low HGS in patients with advanced cancer receiving PC.

**Methods:**

A cross-sectional study was conducted at the Brazilian National Cancer Institute between 2023 and 2025, including patients with advanced cancer receiving PC. HGS was measured using a Jamar® hydraulic dynamometer. HGS values below the first tertile were classified as low HGS. Clinical, sociodemographic, functional, and nutritional data were collected. Analyses, stratified by sex, included logistic regression, with odds ratios (ORs) and 95% confidence intervals (CI) as estimators.

**Results:**

Five hundred twenty-seven patients were included, with a median age of 66 years, predominantly women (58%), and with primary tumor sites in the gastrointestinal tract (24%) and gynecological (21%). The cutoff points defining low HGS (1^st^ tertiles) were: ≤ 12 kg for women and ≤ 20 kg for men. A higher risk of low HGS was observed among older women (OR: 2.29; 95% CI: 1.31–4.01) and those with Karnofsky Performance Status (KPS) 40–50% (OR: 5.06; 95% CI: 3.01–8.49); as well as among older men (OR: 4.02; 95% CI: 2.36–8.35) and those with KPS 40–50% (OR: 3.89; 95% CI: 2.52–6.34).

**Conclusion:**

Advanced age and lower KPS were independently associated with low HGS in patients with advanced cancer receiving palliative care. These findings support HGS as an objective indicator of physical vulnerability in this population.

**Supplementary Information:**

The online version contains supplementary material available at https://doi.org/10.1007/s00520-026-11249-x.

## Introduction

Advanced cancer represents an incurable stage of disease and is frequently associated with marked functional decline, loss of muscle mass, and progressive impairment of physical performance [1, 2]. In this population, such alterations are often exacerbated by cancer-related cachexia, systemic inflammation, anorexia, and the cumulative burden of oncologic treatments, resulting in increased clinical vulnerability and reduced functional autonomy [2–5]. In this context, the assessment of muscle function is essential to monitor the trajectory of functional decline, support clinical decision-making, and identify individuals at greater risk of adverse outcomes. Among the available clinical measures, muscle strength is strongly recommended as a reliable indicator of both nutritional status and functional capacity [6].

Muscle strength can be assessed using handgrip strength (HGS), an objective, practical, and validated measure capable of detecting early changes in muscle function, overall strength, and physical performance [1, 2]. HGS assessment may support the early identification of nutritional and functional impairment and inform timely interventions aimed at delaying or preventing adverse outcomes associated with declining muscle strength as cancer progresses. These outcomes include prolonged hospital stays, poorer quality of life, reduced functional capacity, and shorter survival. In patients with advanced cancer receiving specialized palliative care, HGS may be particularly relevant because it provides an objective assessment of functional capacity, even among frail individuals who are often unable to complete more complex performance-based tests [2, 7–9]. HGS has also been shown to be a more reliable predictor of survival than muscle mass assessed in isolation during advanced stages of cancer. Reductions in HGS may precede measurable declines in muscle mass, thereby enabling earlier detection of functional impairment. Furthermore, muscle mass has been reported to be significantly associated with survival only when accompanied by low HGS [10].

Despite its usefulness, the characterization of HGS in this population remains limited. Most existing studies have focused on community-dwelling older adults or on patients with cancer undergoing active treatment [5, 7, 8, 11], resulting in a scarcity of data specific to the palliative care setting. Moreover, few studies examine factors associated with low HGS in this context [2, 12], and none of them analyze these factors separately for men and women. Such stratification is relevant due to muscular dimorphism, hormonal differences, and distinct trajectories of lean mass loss. An additional challenge is the lack of extensively validated HGS cut-off points for this population, which hampers comparisons across studies and limits clinical interpretation.

In view of these gaps, the present study aimed to evaluate the factors associated with low HGS in patients with advanced cancer receiving palliative care.

## Methods

### Study design

This cross-sectional study was conducted within a broader research project carried out at the Palliative Care Unit (PCU) of a national cancer referral center in Brazil between August 2023 and August 2025. This study was approved by the Institutional Ethics Committee (protocol no. 5.920.862/2023), and all study participants signed the informed consent form. It followed the STROBE recommendations for observation research [13].

### Eligibility criteria

Patients with advanced cancer attending their first outpatient visit to the PCU who met the eligibility criteria were consecutively recruited. The parent project included patients receiving both outpatient and inpatient care and encompassed individuals with a wider range of functional status, including those with Karnofsky Performance Status (KPS) ≥ 30%.

Eligible participants were adults (≥ 20 years) with advanced cancer, defined as metastatic disease confirmed by histological, cytological, or radiological evidence, or as local or locoregional recurrence in patients not undergoing antineoplastic therapy, with KPS ≥ 40% [14]. The KPS threshold of ≥ 40% was established for the present analysis because the study was conducted in an outpatient setting and required participants to be physically capable of completing the HGS assessment. Patients with KPS scores below 40% generally have more severe functional impairment, require substantial assistance with activities of daily living, and are more likely to receive inpatient care. Their clinical condition may also preclude the safe and reliable performance of the standardized HGS test. They also needed the ability to provide the required information, and the absence of severe symptoms that could preclude assessment. Exclusion criteria were the inability to perform HGS measurement for any reason or an HGS value of zero.

### Data collection

#### Dependent variable

Muscle strength was assessed using HGS, measured bilaterally with a Jamar® hydraulic dynamometer (Baseline, Fabrication Enterprises, Inc., USA; range 0–90 kg, resolution 2 kg). Patients were instructed to remain seated with their feet flat on the floor, legs slightly apart, knees flexed at 90°, and arms resting alongside the body. The upper limb being assessed was positioned with the elbow flexed at 90° and the forearm in a neutral position. Each hand was tested three times, alternately, with 1-min intervals between trials [15]. The highest value obtained, regardless of hand dominance, was used for analysis, as supported by Wiegert et al. [16].

HGS was assessed at study enrollment, during the patient’s first outpatient visit to the PCU. All measurements were performed by a trained nutritionist, who was also responsible for collecting the remaining clinical and sociodemographic variables. Consequently, the assessor was not blinded to the patients’ clinical information, including their KPS scores.

For analytical purposes, HGS was categorized as low or non-low using sample-specific, sex-stratified tertiles. Participants in the lowest sex-specific tertile were classified as having low HGS [10, 17]. This approach was adopted because validated HGS cutoff values specifically established for patients with advanced cancer receiving palliative care are currently unavailable. Sex-specific categorization was used to account for well-established differences in muscle strength between women and men and to identify participants with relatively lower HGS within the study population. Although the thresholds proposed by the Asian Working Group for Sarcopenia (AWGS) or the European Working Group on Sarcopenia in Older People (EWGSOP) are commonly used to identify low HGS as part of sarcopenia assessment, they have not been specifically validated in this clinical context [18, 19].

#### Independent variables

Sociodemographic data, including sex, age, self-reported skin color, educational level, total household income, marital status, total household income, and educational level, were collected through interviews. Following the classification of the Brazilian Institute of Geography and Statistics to self-reported skin color, the term *Black* was used to refer to individuals who self-identify as Black or Brown (mixed-race) [20].

Clinical data including primary tumor site, cancer progression, comorbidities, KPS and previous treatments were obtained from medical records. KPS is a semi-objective percentage scale that categorizes patients according to their physical functioning and was used to assess performance status [14].

Nutritional risk was evaluated using first section of the validated Brazilian Portuguese version of the Patient-Generated Subjective Global Assessment (PG-SGA) (©FD Ottery, 2005, 2006, 2015, 2018; Abbott, 2016), available at https://www.pt-global.org/., known as the short form (PG-SGA SF). Nutritional risk was determined based on the total PG-SGA SF score. Patients with a score ≥ 9 points were classified as being at nutritional risk [21–25].

A flowchart outlining the methodological steps of this research can be found in Supplementary Fig. 1.

#### Statistical analysis

Descriptive analyses of the main variables were performed to characterize the sample. The Kolmogorov–Smirnov test was applied to assess data distribution. Categorical variables, including HGS tertiles, were described as absolute frequency (*n*) and relative percentage (%) and compared using the chi-square test.

Both crude and adjusted multiple logistic regression analyses were conducted to evaluate associations with low HGS, with odds ratios (OR) and 95% confidence intervals (95% CI) used as measures of effect. Variables with a *p*-value (*p*) < 0.200 in the crude analyses were selected for inclusion in the adjusted multiple models. In women analysis, the variable diagnoses were included in the adjusted model despite not showing a statistically significant difference, due to its biological plausibility. The final model was constructed using the backward stepwise method. Statistical significance was set at *p* < 0.050.

Data analyses were performed using Stata Data Analysis and Statistical Software, version 15.1 (StataCorp, College Station, TX, USA).

To verify the statistical power of this study, a post hoc test was conducted using the online tool available at https://clincalc.com/Stats/Power.aspx, considering dichotomous outcomes for two independent groups with an alpha error of 0.05. The analysis yielded a statistical power of 96% for women and 90% for men.

## Results

A total of 1209 patients were approached for participation in the broader research project. Of these, 682 were not included in the present analysis because they did not meet the study-specific eligibility criteria or for other reasons detailed in the study flowchart. The final analytical sample comprised 527 participants, of whom 304 (58%) were women and 223 (42%) were men (Supplementary Fig. 2).

The median age of the study population was 66 (interquartile range: 57–74) years (data not shown). The sample consisted predominantly of individuals self-identifying as Black (59%). The most prevalent primary tumor sites were the gastrointestinal tract (24%) and gynecological (21%). The first tertile of HGS was ≤ 12 kg for women and ≤ 20 kg for men (Tables 1 and 2).

**Table 1:** Frequency (%) of sociodemographic variables of patients with advanced cancer in palliative care, according to handgrip strength tertiles, stratified by sex (*n* = 527)

Variables	Total (*n*; %)	HGS					
		Women			Men		
		1 st tertiles (≤ 12 kg)	2nd and 3rd tertiles (> 12 kg)	*p-value*	1 st tertiles (≤ 20 kg)	2nd and 3rd tertiles (> 20 kg)	*p-value*
Age (years)							
< 60	161 (30)	26 (24)	83 (76)	** < 0.001**	8 (15)	44 (85)	** < 0.001**
≥ 60	366 (70)	84 (43)	111 (57)		80 (47)	91 (53)	
Self-reported skin color							
Black^a^	309 (59)	64 (36)	114 (64)	0.921	45 (34)	86 (66)	0.062
Others^b^	218 (41)	46 (36)	80 (64)		43 (47)	49 (53)	
Marital status							
In a marital relationship^c^	245 (46)	28 (27)	77 (73)	**0.012**	66 (47)	74 (53)	**0.002**
Without a marital relationship^d^	282 (54)	82 (41)	117 (59)		22 (26)	61 (74)	
Educational level (years)							
< 9	260 (49)	65 (44)	84 (56)	**0.008**	48 (43)	63 (57)	0.250
≥ 9	267 (51)	45 (29)	110 (71)		40 (36)	72 (64)	
Total household income (minimum wages)							
< 1	161 (31)	34 (33)	70 (67)	0.361	22 (38)	35 (62)	0.877
> 1	366 (69)	76 (38)	124 (62)		66 (40)	100 (60)	

n: number of observations; HGS: handgrip strength

^a^Sum of patients self-identified as brown/*mixed-race* (*n* = 223; 52%) and *Black* (*n* = 86; 16%), and their respective percentages (BRASIL, 2023)

^b^Caucasians, of Asian descent, and of Indigenous descent

^c^Common-law union and married

^d^Single, divorced, and widowed

**Table 2:** Frequency (%) of clinical, functional, and nutritional variables of patients with advanced cancer in palliative care, according to handgrip strength tertiles, stratified by sex (*n* = 527)

Variables	Total (*n*; %)	HGS					
		Women			Men		
		1 st tertiles (≤ 12 kg)	2nd and 3rd tertiles (> 12 kg)	*p*-value	1 st tertiles (≤ 20 kg)	2nd and 3rd tertiles (> 20 kg)	*p*-value
Primary tumor site							
GI tract^a^	126 (24)	23 (41)	33 (59)	0.496	29 (42)	41 (58)	0.898
Gynecological^b^	111 (21)	35 (31)	76 (69)		—	—	
Head and neck^c^	101 (19)	12 (39)	19 (61)		26 (37)	44 (63)	
Breast	37 (7)	13 (35)	24 (65)		—	—	
Skin, bone, and connective tissue	59 (11)	10 (33)	20 (67)		10 (34)	19 (65)	
Lung	32 (6)	12 (55)	10 (45)		5 (50)	5 (50)	
Others^d^	61 (12)	5 (29)	12 (70)		18 (41)	26 (59)	
Cancer progression							
Locally advanced	444 (85)	94 (36)	165 (64)	0.924	69 (37)	116 (63)	0.145
Distant metastatic	451 (85)	96 (36)	169 (64)	0.968	75 (40)	111 (60)	0.555
Previous treatments							
Yes	450 (86)	91 (33)	181 (67)	**0.004**	66 (37)	112 (63)	0.148
No	77 (14)	19 (59)	13 (41)		22 (49)	23 (51)	
Types of previous treatments							
Chemotherapy	362 (69)	69 (30)	157 (70)	** < 0.001**	46 (34)	90 (66)	**0.031**
Surgery	270 (51)	51 (34)	101 (66)	0.340	42 (36)	76 (64)	0.210
Radiotherapy	260 (49)	50 (31)	111 (69)	**0.048**	36 (36)	63 (64)	0.398
Comorbidities							
Yes	380 (72)	94 (41)	135 (59)	**0.002**	62 (41)	89 (59)	0.480
No	147 (28)	16 (21)	59 (79)		26 (36)	46 (64)	
Types of comorbidities							
SAH	260 (49)	66 (43)	89 (57)	**0.018**	45 (43)	60 (57)	0.328
DM	122 (23)	32 (42)	44 (58)	0.215	19 (41)	27 (59)	0.774
CD	53 (10)	16 (57)	12 (43)	**0.015**	13 (52)	12 (48)	0.173
KPS (%)							
40–50	230 (44)	78 (56)	62 (44)	** < 0.001**	55 (61)	35 (39)	** < 0.001**
≥ 60	297 (56)	32 (20)	132 (80)		33 (25)	100 (75)	
PG-SGA SF (score)							
< 9	264 (50)	44 (30)	101 (70)	**0.043**	44 (37)	75 (63)	0.416
≥ 9	263 (50)	66 (41)	93 (59)		44 (42)	60 (58)	

*n* number of observations, *GI tract* gastrointestinal tract, *KPS* Karnofsky Performance Status, *PG-SGA SF* Patient-Generated Subjective Global Assessment Short Form, *HGS* handgrip strength, *DM* diabetes mellitus, *SAH* systemic arterial hypertension, *CD* cardiovascular disease

^a^Gastroesophageal, small intestine, colorectal, liver, pancreas, and bile duct

^b^Cervix, uterus, endometrium, ovary, and vulva

^c^Oral and nasal cavity, pharynx, larynx, salivary glands, paranasal sinuses, and eyes

^d^Bladder, kidney, prostate, neurological, man genital organs, lymphomas, leukemias and myelomas, peritoneum, mediastinum, and unknown primary site

Among women, low HGS was significantly more prevalent among older individuals (≥ 60 years) (*p* < 0.001), those without a marital relationship (*p* = 0.012), and those with fewer than nine years of formal education (*p* = 0.008). Higher frequencies of low HGS were also observed among women who had previously received antineoplastic treatment (*p* = 0.004), particularly chemotherapy (*p* < 0.001) and radiotherapy (*p* = 0.048); had comorbidities (*p* = 0.002), especially hypertension (*p* = 0.018) and cardiovascular disease (*p* = 0.015); KPS ≤ 50% (*p* < 0.001); and PG-SGA SF score ≥ 9 points (*p* = 0.043). Among men, low HGS was more frequent in older individuals (≥ 60 years) (*p* < 0.001), those who self-identify as Black (*p* = 0.062), and those with a partner (*p* = 0.002). Additionally, low HGS was more frequent among men who had previously received chemotherapy (*p* = 0.031) and those with KPS ≤ 50% (*p* < 0.001) (Tables 1 and 2).

For women, the variables showing statistically significant differences in the crude model were age (OR 2.41; 95% CI 1.43–4.07), educational level (OR 1.89; CI 95% 1.17–3.03), KPS (OR 5.18; 95% CI 3.11–8.64), hypertension (OR 1.76; 95% CI 1.10–2.84), cardiovascular disease (OR 2.58; 95% CI 1.17–5.68), and PG-SGA SF (OR 1.62; 95% CI 1.01–2.61) (Table 3). In the adjusted model (R^2^: 13%), only age ≥ 60 years (OR 2.29; 95% CI 1.31–4.01), and KPS ≤ 50% (OR 5.06; 95% CI 3.01–8.49) remained associated with low HGS (Table 4).

**Table 3:** Univariate logistic regression of factors associated with low handgrip strength in patients with advanced cancer in palliative care, stratified by sex (*n* = 527)

Variables	Women		Men	
	OR (95% CI)	***p***-value	OR (95% CI)	***p***-value
Age (years)				
< 60	ref		ref	
≥ 60	2.41 (1.43; 4.07)	** < 0.001**	4.83 (2.14; 10.87)	** < 0.001**
Self-reported skin color				
Others^a^	1.02 (0.63; 1.64)	0.921	1.67 (0.97; 2.89)	0.063
Black^b^	ref		ref	
Educational level (years)				
< 9	1.89 (1.17; 3.03)	**0.008**	1.37 (0.80; 2.35)	0.251
≥ 9	ref		ref	
KPS (%)				
40–50	5.18 (3.11; 8.64)	** < 0.001**	4.76 (2.67; 8.49)	** < 0.001**
≥ 60	ref		ref	
Primary tumor site				
GI tract^c^	1.67 (0.51; 5.39)	0.389	1.19 (0.60; 2.36)	0.604
Gynecological^d^	1.10 (0.36; 3.37)	0.861	—	
Head and neck^e^	1.51 (0.42; 5.39)	0.521	ref	
Breast	1. 3 (0.37; 4.50)	0.679	—	
Skin, bone and connective tissue	1.2 (0.33; 4.36)	0.782	0.89 (0.35; 2.20)	0.802
Lung	2.88 (0.75; 10.98)	0.122	1.69 (0.44; 6.40)	0.439
Others^f^	ref		1.17 (0.54; 2.53)	0.688
DM				
Yes	1.39 (0.82; 2.37)	0.216	1.10 (0.56; 2.13)	0.774
No	ref		ref	
SAH				
Yes	1.76 (1.10; 2.84)	**0.018**	1.30 (0.76; 2.24)	0.328
No	ref		ref	
CD				
Yes	2.58 (1.17; 5.68)	**0.018**	1.77 (0.77; 4.09)	0.178
No	ref		ref	
PG-SGA SF (score)				
< 9	ref		ref	
> 9	1.62 (1.01; 2.61)	**0.044**	1.25 (0.72; 2.14)	0.417

*ref.* reference, *PG-SGA SF* Patient-Generated Subjective Global Assessment Short Form, *KPS* Karnofsky Performance Status, *DM* diabetes mellitus, *SAH* systemic arterial hypertension, *CD* cardiovascular disease, *OR* odds ratio, *CI* confidence interval

^a^Caucasians, of Asian descent, and of Indigenous descent

^b^Sum of patients self-identified as *brown*/*mixed-race* (*n* = 223; 42%) and *Black* (*n* = 86; 16%) and their respective percentages (BRASIL, 2023)

^c^Gastroesophageal, small intestine, colorectal, liver, pancreas, and bile duct

^d^Cervix, uterus, endometrium, ovary, and vulva

^e^Oral and nasal cavity, pharynx, larynx, salivary glands, paranasal sinuses, and eyes

^f^Bladder, kidney, prostate, neurological, man genital organs, lymphomas, leukemias and myelomas, peritoneum, mediastinum, and unknown primary site

**Table 4:** Final model of factors associated with low handgrip strength in patients with advanced cancer in palliative care, stratified by sex (*n* = 527)

Variables	Women		Men	
	OR (95% CI)	***p***-value	OR (95% CI)	***p***-value
Age (years)				
< 60	ref		ref	
≥ 60	2.29 (1.31; 4.01)	**0.004**	4.02 (2.36; 8.35)	** < 0.001**
KPS (%)				
40–50	5.06 (3.01; 8.49)	** < 0.001**	3.89 (2.52; 6.34)	** < 0.001**
≥ 60	ref		ref	

*ref.* reference, *KPS* Karnofsky Performance Status, *OR* odds ratio, *CI* confidence interval

For men, the variables showing statistically significant differences in the crude model were age (OR 4.83; 95% CI 2.14–10.87) and KPS (OR 4.76; 95% CI 2.67–8.49) (Table 3). In the adjusted model (R^2^: 16%), the variables that remained associated with low HGS were age ≥ 60 years (OR 4.02; 95% CI 2.36–8.35), and KPS ≤ 50% (OR 3.89; 95% CI 2.52; 6.34) (Table 4).

The distribution of low HGS according to age (> 60 years) and KPS categories, stratified by sex, is presented in Supplementary Table 1, and the proportion of low HGS within each KPS category, stratified by sex, is presented in Supplementary Table 2.

## Discussion

In this large cohort of adults with advanced cancer receiving specialized palliative care, older age and poorer functional performance emerged as the main factors associated with low HGS in both women and men. These findings support the hypothesis that age and functional characteristics are closely related to muscle strength in advanced disease and reinforce the potential relevance of HGS as an objective indicator of physical vulnerability in palliative care.

The association between advancing age and lower HGS is well documented in both general and geriatric populations, reflecting the progressive decline in muscle mass and strength that occurs with aging. In patients with advanced cancer, this decline may be further exacerbated by cachexia, systemic inflammation, anorexia, and the increased metabolic demands imposed by the disease, all of which may contribute to accelerated muscle loss and sarcopenia progression [26, 27]. These changes have been associated with poorer clinical outcomes, including reduced overall survival. Accordingly, the association between older age and low HGS observed in the present study may reflect the combined effects of age-related muscle decline and the catabolic burden of advanced cancer [27].

Functional performance, assessed using the KPS, emerged as one of the factors most strongly associated with low HGS in both sexes. It is well established that patients with KPS between 40% and 50% experience substantial limitations in activities of daily living and increased levels of dependency, reflecting reduced overall functional capacity. The close convergence between low HGS and reduced KPS observed in this study suggests that muscle strength assessment may provide a rapid and objective approximation of global functional status. Although the cross-sectional design of this study precludes causal inferences, a bidirectional relationship is biologically and clinically plausible, whereby functional decline leads to reduced muscle use and strength, while diminished muscle strength may, in turn, accelerate loss of autonomy and functional deterioration.

The association identified in this study is supported by previous evidence linking HGS with functional decline and adverse clinical outcomes. Taekema et al. [11], conducted with older adults aged 85 and 89 years from Leiden, observed that low HGS was associated with poorer functional scores and predicted the decline of daily activities. Similarly, Kilgour et al. [2], investigating HGS as a predictor of survival and its relationship with clinical and functional markers in patients with advanced cancer, demonstrated that HGS may serve as an important clinical proxy. In their study, patients with HGS at or below the 10th percentile exhibited poorer performance status, reduced lean mass, increased levels of fatigue, worse quality of life, higher prevalence of sarcopenia, poorer survival, lower body mass index, and reduced hemoglobin and albumin levels.

More recently, Hadzibegovic et al. [28], in a prospective study including predominantly patients with advanced cancer, demonstrated that lower HGS was associated with poorer functional status and physical performance and independently associated with increased mortality. Sakaguchi et al. [5] further showed that low HGS was associated with multiple indicators of disease severity, including poorer performance status, nutritional impairment, systemic inflammation, and reduced muscle reserves, supporting the concept that HGS may reflect a broader phenotype of clinical vulnerability. In addition, Fukushima et al. [29] found that muscle strength measures, including HGS, were associated with survival in patients with advanced or recurrent lung cancer, whereas skeletal muscle mass alone was not. Taken together, these findings reinforce the clinical relevance of muscle function assessment in advanced cancer. Although prognosis was not assessed in the present study, our findings extend this evidence by showing that KPS < 50% was strongly and independently associated with low HGS in both women and men.

The present findings may have relevant implications for clinical practice in oncologic palliative care. First, HGS emerges as a simple, low-cost tool that can be easily incorporated into routine care and provides an objective measure of overall functional capacity. Identifying patients with low HGS may support decisions related to care planning, risk stratification, and resource allocation, particularly in settings where more complex functional assessments are not feasible. Furthermore, the strong association between low HGS, advanced age, and reduced KPS suggests that assessing muscle strength may help to identify, at an early stage, individuals with heightened clinical vulnerability, enabling more timely interventions in areas such as nutrition, symptom management, and palliative rehabilitation.

Notably, previous studies have shown that HGS can detect changes in muscle function even in patients who still exhibit good performance status [27]. Importantly, although age and KPS were independently associated with low HGS, their explanatory power was limited, accounting for only 13–16% of the variability. Overall, 36% of women and 31% of men were classified as having low HGS, whereas the corresponding prevalence among patients aged > 60 years with KPS scores ≤ 50% increased substantially to 63% in women and 71% in men. Therefore, these variables alone are insufficient to identify all patients with low HGS, but they may still serve as useful screening indicators for early detection of individuals at risk of muscle loss.

From a clinical perspective, the present findings suggest that age and KPS may serve as accessible indicators of increased vulnerability to reduced muscle strength in patients with advanced cancer receiving palliative care. Although these variables explained only a modest proportion of the variability in HGS, their consistent association across sexes highlights their potential role in early risk awareness within routine clinical assessment.

Importantly, HGS measurement remains the most appropriate method for objectively assessing muscle strength and should be performed whenever feasible. However, in contexts where dynamometry is not readily available, the combined consideration of older age and lower KPS may help clinicians identify individuals who warrant closer functional monitoring and more comprehensive evaluation. Such an approach may facilitate timely, proportionate supportive strategies while acknowledging the multifactorial nature of muscle weakness in advanced disease.

Reference values for HGS derived from healthy populations may not be directly applicable to patients with advanced cancer, whose muscle strength is frequently affected by disease-related catabolism, functional impairment, and muscle mass depletion. A previous study involving patients with cancer receiving palliative care also used the lowest sex-specific tertile of the study sample to define low HGS and reported cutoff values of < 20.0 kg for men and < 13.0 kg for women [10]. These values are very similar to those identified in the present study (< 20.0 kg for men and < 12.0 kg for women), suggesting consistency across comparable clinical populations. Nevertheless, because both studies used sample-specific tertiles, this similarity should not be interpreted as external validation of a diagnostic threshold. Although previous studies have demonstrated the prognostic value of HGS in advanced cancer, prognosis was not assessed in the present study. Prospective studies are therefore needed to determine whether the sex-specific HGS thresholds identified here are associated with survival and other clinically relevant outcomes.

This study has several strengths that deserve emphasis: a large sample size for the palliative care context, standardized HGS measurements, sex-stratified analyses, and the incorporation of widely validated functional and nutritional indicators. The fact that it was conducted in a national referral center ensures rigor in the assessments, although it may limit the generalizability of the findings to settings with fewer available resources. We also highlight that this is the first study to evaluate factors associated with low HGS in this context, contributing to the identification of individuals at higher risk of presenting low HGS and supporting early interventions.

However, some limitations should be considered. Our sample was predominantly composed of older adults, making it difficult to apply the results to younger patients with advanced cancer receiving palliative care. Furthermore, the cross-sectional nature of the study prevents us from inferring causality. The relatively large number of patients not included in the present analysis reflects the broader scope of the research project, which enrolled both outpatients and inpatients, including individuals with KPS scores below the eligibility threshold adopted for this study. As the present analysis included only outpatients with KPS scores ≥ 40% who were able to complete the HGS assessment, the final sample represented a more functionally preserved population. Consequently, the findings may not be generalizable to hospitalized patients, individuals with KPS scores < 40%, or those with more severe functional impairment.

Low HGS was defined using sample-specific, sex-stratified tertiles rather than established diagnostic cutoff values. This approach was adopted because validated HGS thresholds specifically established for patients with advanced cancer receiving palliative care are currently unavailable, and muscle strength in this population may be substantially affected by advanced disease, muscle mass depletion, and functional impairment. However, the use of sample-specific tertiles limits direct comparability with studies applying the AWGS or EWGSOP criteria and does not allow participants to be classified according to an externally validated threshold for low HGS. Therefore, the findings should be interpreted as identifying relatively low HGS within the study population rather than establishing an absolute clinical classification.

Another aspect to consider is that the analog hydraulic Jamar® dynamometer requires a minimum force of 3–4 kg to move the gauge and allow HGS measurement, which may compromise assessments in patients who are extremely frail, sarcopenic, or cachectic. In this sense, the exclusion of patients with an HGS value of zero (due to the technical impossibility of activating the device) may have introduced a slight selection bias, potentially underestimating the magnitude of muscle weakness among the most vulnerable individuals. Even so, this decision was necessary to ensure measurement accuracy and avoid misinterpretation. Nonetheless, HGS remains a validated and highly reliable method for use in patients with advanced cancer [30, 31]. In addition, HGS measurements and the collection of the remaining study variables were performed by the same researcher, who was not blinded to the patients’ clinical information, including KPS scores. Although HGS was measured using a standardized protocol, the lack of assessor blinding may have introduced measurement or observer bias.

Despite its potential, the use of HGS in palliative care still lacks broad standardization and validation. Longitudinal studies are awaited to investigate the temporal relationship between low HGS and functional decline, aiming to determine whether low HGS can be detected before functional deterioration, or even whether a decrease in KPS can serve as an early indicator of muscle function decline.

Pragmatic clinical trials are also needed to evaluate whether targeted interventions, such as intensive nutritional support, specialized physical therapy, or multimodal rehabilitation strategies, can modify muscle strength or mitigate its effects on the functional trajectory of these patients. Finally, multicenter and international approaches may broaden the generalizability of findings and contribute to the establishment of evidence-based guidelines for the use of HGS in oncologic palliative care.

## Conclusion

Advanced age and lower KPS were independently associated with low HGS in patients with advanced cancer receiving palliative care. These findings support HGS as an objective indicator of physical vulnerability in this population.

## Supplementary Information

Below is the link to the electronic supplementary material.

ESM 1 (DOCX 15.2 KB)

ESM 2 (DOCX 102 KB)

## Data Availability

The data and analysis material related to this study are maintained and managed according to ethical regulations. To maintain the confidentiality and anonymity of patients, this information will not be made publicly available. Requests for further information can be directed to the corresponding author.
