# Supplementary material for: Low handgrip strength in advanced cancer: associated factors and clinical implications in palliative care
Source: Support Care Cancer. 2026 Sep 26;34(10):1020. doi: 10.1007/s00520-026-11249-x (PMC13615993; doi:10.1007/s00520-026-11249-x)
Supplement: Supplementary file 1 [file 520_2026_11249_MOESM1_ESM.docx]

**Supplementary Table 1.** Frequency of low handgrip strength, according to Karnofsky Performance Status in patients over 60 years old with advanced cancer in palliative care, stratified by sex (n= 164).

|  | **Low HGS*** | |
| --- | --- | --- |
| **KPS (%)** | **Women**  **n (%)** | **Men**  **n (%)** |
| >50 | 23 (23) | 29 (29) |
| ≤50 | 61 (63) | 51 (71) |
| **Total** | 84 (43) | 80 (47) |

Note: n: number of observations; HGS: handgrip strength; KPS: Karnofsky Performance Status.

*low HGS=1st tertile .

**Supplementary Table 2.** Frequency of handgrip strength tertiles, according to Karnofsky Performance Status in patients with advanced cancer in palliative care, stratified by sex (n= 527).

| **KPS (%)** | **HGS Women**  **(n;%)** | | **p-value** | **HGS Men  (n;%)** | | **p-value** |
| --- | --- | --- | --- | --- | --- | --- |
|  | **1st tertiles***  (n ; %) | **2nd e 3rd tertiles**  (n ; %) |  | **1st tertiles***  (n ; %) | **2nd e 3rd tertiles**  (n ; %) |  |
| 40 | 38 (68) | 18 (32) | **<0.001** | 23 (68) | 11 (32) | **<0.001** |
| 50 | 40 (48) | 44 (52) |  | 32 (57) | 24 (43) |  |
| 60 | 21 (23) | 68 (76) |  | 18 (30) | 41 (70) |  |
| 70 | 7 (16) | 35 (83) |  | 11 (21) | 41 (79) |  |
| 80 | 4 (15) | 23 (85) |  | 4 (26) | 11 (74) |  |
| 90 | - | 4 (100) |  | - | 7 (100) |  |
| 100 | - | 2 (100) |  | - | - |  |

Note: n: number of observations; HGS: handgrip strength; KPS: Karnofsky Performance Status;

*1st tertile = low HGS.
