# Supplementary material for: Low handgrip strength in advanced cancer: associated factors and clinical implications in palliative care
Source: Support Care Cancer. 2026 Sep 26;34(10):1020. doi: 10.1007/s00520-026-11249-x (PMC13615993; doi:10.1007/s00520-026-11249-x)
Supplement: Supplementary file 2 [file 520_2026_11249_MOESM2_ESM.docx]

**SUPPLEMENTARY MATERIAL**

**Supplementary Figure 1** **–** Flowchart outlining the methodological steps of this article.

Note: KPS - Karnofsky performance status; HGS - Handgrip strength; PG-SGA SF - Patient-Generated Subjective Global Assessment short form.

**Supplementary Figure 2 –** Flowchart of participants’ selection.

Note: KPS – Karnofsky Performance Status; HGS – Handgrip strength.

*Symptoms such as dyspnea, pain, emotional conditions, communication difficulties, mental confusion, nausea, vomiting, fatigue, among others.

**Other reasons: For instance, being in consultation at the time, which prevented the team from collecting the data
